# Vascular Cognitive Impairment in a Memory Clinic Population: Rationale and Design of the “Utrecht-Amsterdam Clinical Features and Prognosis in Vascular Cognitive Impairment” (TRACE-VCI) Study

**DOI:** 10.2196/resprot.6864

**Published:** 2017-04-19

**Authors:** Jooske Marije Funke Boomsma, Lieza Geertje Exalto, Frederik Barkhof, Esther van den Berg, Jeroen de Bresser, Rutger Heinen, Huiberdina Lena Koek, Niels Daniël Prins, Philip Scheltens, Henry Chanoch Weinstein, Wiesje Maria van der Flier, Geert Jan Biessels

**Affiliations:** ^1^ Department of Neurology, Neurosurgery and Neuropsychology, Brain Centre Rudolf Magnus Institute University Medical Centre Utrecht Utrecht Netherlands; ^2^ Onze Lieve Vrouwe Gasthuis (OLVG) West Department of Neurology Amsterdam Netherlands; ^3^ Department of Radiology & Nuclear Medicine VU University Medical Centre Amsterdam Netherlands; ^4^ Department of Neurology Erasmus MC University Medical Centre Rotterdam Netherlands; ^5^ Department of Radiology University Medical Centre Utrecht Utrecht Netherlands; ^6^ Department of Geriatrics University Medical Centre Utrecht Utrecht Netherlands; ^7^ Alzheimer Centre and Department of Neurology VU University Medical Centre Amsterdam Netherlands; ^8^ Department of Epidemiology and Biostatistics VU University Medical Centre Amsterdam Netherlands

**Keywords:** vascular cognitive impairment, memory clinic, small vessel disease, vascular disease, prognosis, dementia

## Abstract

**Background:**

Vascular Cognitive Impairment (VCI) refers to cognitive dysfunction due to vascular brain injury, as a single cause or in combination with other, often neurodegenerative, etiologies. VCI is a broad construct that captures a heterogeneous patient population both in terms of cognitive and noncognitive symptoms and in terms of etiology and prognosis. This provides a challenge when applying this construct in clinical practice.

**Objective:**

This paper presents the rationale and design of the TRACE-VCI study, which investigates the clinical features and prognosis of VCI in a memory clinic setting.

**Methods:**

The TRACE-VCI project is an observational, prospective cohort study of 861 consecutive memory clinic patients with possible VCI. Between 2009 and 2013, patients were recruited through the Amsterdam Dementia Cohort of the VU University Medical Centre (VUMC) (N=665) and the outpatient memory clinic and VCI cohort of the University Medical Centre Utrecht (UMCU) (N=196). We included all patients attending the clinics with magnetic resonance imaging (MRI) evidence of vascular brain injury. Patients with a primary etiology other than vascular brain injury or neurodegeneration were excluded. Patients underwent an extensive 1-day memory clinic evaluation including an interview, physical and neurological examination, assessment of biomarkers (including those for Alzheimer-type pathologies), extensive neuropsychological testing, and an MRI scan of the brain. For prognostic analyses, the composite primary outcome measure was defined as accelerated cognitive decline (change of clinical dementia rating ≥1 or institutionalization) or (recurrent) major vascular events or death over the course of 2 years.

**Results:**

The mean age at baseline was 67.7 (SD 8.5) years and 46.3% of patients (399/861) were female. At baseline, the median Clinical Dementia Rating was 0.5 (interquartile range [IQR] 0.5-1.0) and the median Mini-Mental State Examination score was 25 (IQR 22-28). The clinical diagnosis at baseline was dementia in 52.4% of patients (451/861), mild cognitive impairment in 24.6% (212/861), and no objective cognitive impairment in the remaining 23.0% (198/861).

**Conclusions:**

The TRACE-VCI study represents a large cohort of well-characterized patients with VCI in a memory clinic setting. Data processing and collection for follow-up are currently being completed. The TRACE-VCI study will provide insight into the clinical features of memory clinic patients that meet VCI criteria and establish key prognostic factors for further cognitive decline and (recurrent) major vascular events.

## Introduction

Cerebral vascular injury is a common cause of dementia and milder forms of cognitive dysfunction [1]. This vascular burden in cognitive decline and dementia is referred to as vascular cognitive impairment (VCI) [2,3]. According to the current literature, the concept or construct of VCI covers the entire spectrum of cognitive disorders ranging from mild cognitive impairment (MCI) through fully developed dementia, due to all forms of vascular brain injury [4]. It includes vascular disease as a single etiology, but also in combination with other, often neurodegenerative, causes of cognitive impairment [3,4].

VCI is thus a broad construct that captures a heterogeneous patient population both in terms of cognitive and noncognitive symptoms and in terms of etiology and prognosis. In clinical practice, VCI is mainly observed in stroke (in- and outpatient) services and in memory clinics (ie, outpatient cognitive impairment and dementia diagnostic centers). In a stroke clinic setting, mild to severe cognitive deficits may manifest themselves acutely or delayed after an ischemic or hemorrhagic stroke, in which case it is generally straightforward to establish a causal link between the vascular event and the cognitive deficit. In a memory clinic setting, patients more often present with insidious cognitive changes evolving over the course of many years. Vascular injury in these patients most commonly involves small vessel disease [5]. In many cases, multiple vascular lesions co-exist and often also co-occur with neurodegenerative pathologies, in particular Alzheimer’s disease [3,4]. These mixed pathologies can make it more challenging to establish causality between the vascular lesions and the cognitive deficits in individual patients. In fact, there are no validated and generally accepted thresholds at which visible vascular brain injury can be considered “clinically relevant” in a patient presenting at a memory clinic. Moreover, it is still unclear to which extent vascular injury determines prognosis in a memory clinic setting, particularly when it co-occurs with other etiologies [6]. Addressing these uncertainties is important, given the frequent occurrence of vascular lesions in memory clinic patients. Yet, there are few studies on VCI in memory clinic cohorts [7].

The overall aim of the “Utrecht-Amsterdam clinical features and prognosis in vascular cognitive impairment” (TRACE-VCI) study is to establish the relation between different patterns of cerebral vascular injury and the cognitive profile and prognosis of patients in a memory clinic setting. We want to establish which clinical features of patients cluster in particular VCI phenotypes. In addition, we aim to identify key prognostic factors for further cognitive decline and/or (recurrent) major vascular events. To this end, we prospectively collected data of all patients with cognitive complaints and any burden of vascular brain injury on magnetic resonance imaging (MRI) from our memory clinics between 2009 and 2013. When this study was initiated, the construct of VCI had been introduced [2,3], but widely accepted clinical criteria for VCI were still lacking. In the absence of applicable VCI criteria, we intentionally developed nonrestrictive inclusion criteria for our study. We did not define minimal thresholds for cognitive impairment or specific patterns of vascular brain injury, in order to capture the whole spectrum of patients presenting at a memory clinic with cognitive complaints and visible vascular injury on MRI. We also did not select patients based on evidence for absence or presence of other neurodegenerative etiologies. This approach allowed us to study the full spectrum of cognitive disorders in relation to vascular brain injury as seen in a memory clinic and detect critical thresholds for clinically relevant vascular injury in this setting. Further subdivisions or selections according to stages of cognitive impairment, specific burden of vascular injury, and co-occurring pathologies will be applied as part of the analytic strategy of the TRACE-VCI study. This paper describes the design and protocol of the study including the baseline characteristics of the study population.

## Methods/Design

### Study Design

The TRACE-VCI study is a prospective observational follow-up study of 861 consecutive memory clinic patients from three Dutch outpatient clinics at two university hospitals. These tertiary referral clinics receive referrals from specialists from other memory clinics (eg, for a second opinion) but also direct referrals from general practitioners. Subjects were included from the outpatient clinic of the VU University Medical Centre (VUMC), registered in the Amsterdam Dementia Cohort (N=665) and from the two outpatient memory clinics of the University Medical Centre Utrecht (UMCU) (N=196) [8,9]. Patients with cognitive complaints and any burden of vascular brain injury on MRI (inclusion criteria are specified below) were prospectively included at their first visit to the clinics between September 2009 and December 2013. Each patient received a standardized extensive 1-day memory clinic evaluation including an interview, physical and neurological examination, laboratory testing, extensive neuropsychological testing, and an MRI scan of the brain [4]. The study was approved by the institutional review board of the VUMC and the UMCU. All patients provided informed consent prior to research related procedures.

Follow-up investigation was performed around 2 years from baseline visit. Primary outcome variable for the prognostic studies was cognitive decline and/or (recurrent) major vascular events. These outcome variables are described in detail in the follow-up investigation section.

### Study Objectives

The main objectives of the TRACE-VCI study are:

1. To establish the clinical features of memory clinic patients with vascular brain injury on MRI, addressing the following questions:
  - What are the patterns of vascular brain injury on MRI?
  - What are the cognitive profiles of the patients and how does the nature and severity of vascular brain injury relate to these profiles?
  - How does vascular brain injury relate to noncognitive outcomes, for example, depression, gait, and falls?
  - How do vascular brain injury and co-existent neurodegenerative disease interact?
  - Which features cluster in particular VCI phenotypes?
2. To identify key prognostic factors in patients with possible VCI at a memory clinic:
  - Which factors predict further cognitive decline or (recurrent) major vascular events?
  - Can poor outcome be reliably predicted on an individual basis?

### Inclusion Criteria

We included patients with possible VCI with minimal constraints in terms of cognitive impairment and vascular brain injury. The presence of other co-occurring etiologies, such as neurodegenerative disease or depression was accepted because many patients with VCI have neurodegenerative disease as a comorbid etiology and depression can be a manifestation of cerebrovascular disease [3,10]. According to this rationale, possible VCI was defined according to the following criteria in the TRACE-VCI study.

#### Cognitive Impairment

Patients were included in the TRACE-VCI study regardless of the severity of cognitive impairment. The only criterion was that people had to be referred to the memory clinic because of suspected cognitive impairment. Patients were divided in three categories related to the extent of cognitive impairment: dementia, mild cognitive impairment (MCI), and a third group with no objective cognitive impairment (NOCI). The rationale for including patients with NOCI (also referred to as subjective cognitive impairment in memory clinics [11]) is that some patients with cognitive complaints and cognitive dysfunction due to vascular brain injury may not meet formal criteria for cognitive impairment on psychometric testing. We therefore decided to include all patients with cognitive complaints and evidence of vascular brain injury in the cohort and address different categories of cognitive impairment in the analyses.

#### Vascular Brain Injury

Any patient with at least a minimal burden of vascular brain injury on MRI was eligible for TRACE-VCI. As for cognition, we deliberately did not specify a threshold of injury. Moreover, we included patients regardless of the judgment of the treating physician on the clinical relevance of the vascular brain injury. To be included in TRACE-VCI, patients had to show at least one of the following forms of vascular brain injury on MRI, rated according to established criteria (Table 1) [12]:

1. white matter hyperintensities (WMH) with a Fazekas scale [13] grade ≥2
2. Fazekas scale grade 1 and an increased vascular risk defined as the presence of ≥2 vascular risk factors (hypertension, hypercholesterolemia, diabetes mellitus, obesity, current smoking, or a reported history of a vascular event other than stroke)
3. ≥1 lacunar infarct(s)
4. ≥1 nonlacunar infarct(s)
5. ≥1 cerebral microbleed(s)
6. ≥1 intracerebral hemorrhage

**Table 1:** Entry criteria for vascular brain injury^a^.

Vascular brain injury	Mixed or single vascular injury, n (N=861)	Single vascular injury only, n (N=510)
Fazekas score 1 and ≥2 vascular risk factors	307	210
Fazekas score 2 or 3	399	160
≥1 Lacunar infarct(s)	188	13
≥1 Nonlacunar infarct(s)	83	4
≥1 Microbleed(s) (N=849)	368	120
≥1 Intracerebral hemorrhage(s)	16	3

^a^If there were missing data, the number (n=) is specifically mentioned.The first column presents the proportion of patients meeting the different entry criteria of vascular brain injury, either as a single criterion or in combination with others. The second column lists only patients who presented with a single category of vascular brain injury markers.

For criterion 2, the presence of hypertension was determined based on a self-reported medical history, use of antihypertensive drugs, or a newly diagnosed hypertension defined as a blood pressure of 140/90 mmHg or more, measured by means of a sphygmomanometer [14]. Hypercholesterolemia was determined based on medical history or medication use. Diabetes mellitus was based on medical history or medication use. Glucose or HbA1c levels were available from 96.9% (834/861) of patients. Patients were classified as newly diagnosed diabetes mellitus if they had a nonfasting glucose of ≥11.1 mmol/l or an HbA1c ≥48 mmol/mol (or ≥6.5%) [15]. Obesity was defined as a baseline body mass index (BMI) ≥30, calculated as weight in kilograms divided by height in meters squared. A self-reported history of a vascular event other than stroke was defined as a history of ischemic heart disease (myocardial infarction, surgery, or endovascular treatment for coronary artery disease) [16], peripheral arterial disease (any arterial occlusion or surgical intervention of a peripheral artery such as an abdominal or leg artery), and carotid artery stenting.

### Exclusion Criteria

Patients with a monogenic nonvascular or vascular cause of cognitive dysfunction were excluded from the study population. These genetic diseases are relatively rare and have a distinct disease profile, which is in many aspects different from the other patients in this cohort. Patients with other nonvascular and nondegenerative primary causes of cognitive dysfunction such as a brain tumor, extensive traumatic head injury, substance or alcohol abuse, and multiple sclerosis were also excluded. Finally, patients with psychiatric diseases, other than depression, resulting in cognitive dysfunction were excluded.

### Interview and Physical and Neurological Examination

Patients received a standardized diagnostic assessment performed by a neurologist or geriatrician including an interview on cognitive complaints and medical history, medication use (verified through listings provided by pharmacy), educational level, smoking, alcohol and drug abuse, family medical history, and social status. Patients were asked to bring a relative or good friend for an informant interview. Table 2 shows demographic characteristics and vascular risk factors of the study population.

**Table 2:** Demographic characteristics and vascular risk factors in the TRACE-VCI study.

			Patients (N=861)
**Demographic characteristics**			
	Female, n (%)		399 (46.3)
	Age in years, mean (SD)		67.7 (8.5)
	Level of education (Verhage scale range 1-7)^a^ (N=856), median (IQR)		5 (4-6)
**Vascular risk factors, n (%)**			
	**Hypertension**		729 (84.7)
		Medical history/use of medication	499 (68.4)
		Newly diagnosed hypertension (>140/90mmHg) (N=834)	230 (31.6)
	Hypercholesterolemia		386 (44.8)
	**Diabetes mellitus**		169 (19.6)
		Medical history/ use of medication	146 (86.4)
		Newly diagnosed diabetes mellitus (N=834)	23 (13.6)
	Current smoker (N=853)		173 (20.1)
	Obesity (BMI ≥30) (N=848)		176 (20.4)
**History of reported vascular events, n (%)**			
	History of reported stroke		78 (9.1)
	**History of reported vascular events other than stroke**		86 (10.0)
		History of ischemic heart disease^b^	60 (69.8)
		History of carotid artery stenting	4 (4.7)
		History of peripheral arterial disease^c^	31 (36.0)

^a^Verhage scale: (1) <6 years of primary education, (2) finished 6 years of primary education, (3) 6 years primary education and <2 years of low level secondary education, (4) 4 years of low level secondary education, (5) 4 years of average level secondary education, (6) 5 years of high level secondary education, (7) university degree [17].

^b^Myocardial infarction, surgery or endovascular treatment for coronary artery disease [16].

^c^Any arterial occlusion or surgical intervention of a peripheral artery (eg, abdominal or leg artery).

Physical examination included blood pressure measurement, height (centimeters), weight (kilograms), and BMI. Neurological examination was performed with special attention for higher cortical functions, focal deficits, extrapyramidal signs, balance, gait, primitive reflexes, and postural reaction.

### Cognitive Assessment

#### Cognitive Screening and Education

We used the Dutch version of the Mini-Mental State Examination (MMSE; maximum score of 30) as a cognitive screening test [18]. Furthermore, the cognitive and self-contained part of the Cambridge Examination for Mental Disorders of the Elderly (CAMCOG; maximum score of 107) was performed [19]. Level of education was defined according to a 7-point rating scale (Verhage scale 1-7; low to high education) [17]. The severity of cognitive symptoms was assessed using the Clinical Dementia Rating (CDR; 0-3) global score [20].

#### Psychological Assessment and Other Questionnaires

Neuropsychiatric and behavioral symptoms were evaluated by the 15-item Geriatric Depression Scale (GDS) [21] and the Neuropsychiatric Inventory (NPI; maximum score of 144) [22]. The Disability Assessment for Dementia (DAD; maximum score of 100) questionnaire investigated functional decline [23]. The NPI and DAD were collected through the use of a proxy-respondent. Table 3 shows the cognitive and psychological screening scores at baseline.

**Table 3:** Cognitive and psychological assessment in the TRACE-VCI population (N=861).

Instruments/methods		n (%)		Median (IQR)	
**Global functioning**					
	DAD		738 (85.7)		89 (75-98)
**Mood**					
	GDS		815 (94.7)		3 (2-5)
	NPI^a^		604 (70.2)		10 (4-19)
**Measures of global cognitive status**					
	CDR		861 (100)		0.5 (0.5-1)
	MMSE score		856 (99.4)		25 (22-28)
	CAMCOG^b^		698 (81.1)^c^		82 (69-91)

^a^A higher NPI score relates to more neuropsychiatric symptoms.

^b^Reference values of the CAMCOG score depend on primary education level and age.

^c^The outpatient memory clinic of the UMCU did not perform the CAMCOG, and the VCI outpatient clinic of the UMCU introduced it at a later stage; therefore, 163 (18.9%) were missing

#### Neuropsychological Assessment

All participants performed an extensive neuropsychological examination, with some variation between the centers and over time. This battery has been established through a Dutch multicenter university memory clinic research program on diagnosis and prognosis of cognitive impairment and dementia [8]. Only tasks that were available by the majority of patients (>80%) were included. The tasks were summarized in five major widely used cognitive domains to reduce the amount of neuropsychological variables for statistical analysis and clinical interpretation: (1) working memory, (2) memory, (3) attention and executive functioning, (4) processing speed, and (5) perception and construction. The tests included in each of the domains are listed in Table 4.

The domain working memory was assessed by the Digit Span of the Wechsler Adult Intelligence Scale – 3^rd^ edition (WAIS-III). Patients were asked to verbally repeat series of digits of increasing length in forward and backward condition.

The domain memory was assessed by the Dutch version of the Rey Auditory Verbal Learning Test (RAVLT). For the RAVLT, the total number of words remembered in five learning trials was recorded and the delayed recall and recognition tasks were used. Furthermore, the Visual Association Test (VAT) part A was included to assess visuospatial association learning.

The domain attention and executive functioning was assessed using the ratio of the Trail Making Test part B and A (TMT-B and TMT-A), the Stroop Color Word Test, and the category naming tasks (animal naming, 1 minute) and lexical fluency tasks (letters, 1 minute). The used letters in the lexical fluency tasks were different between the clinics. The letters “N” and “A” were used in 66 patients of the VCI outpatient clinic UMCU. The letters “D,” “A,” and “T” were used in 626 patients of the Amsterdam Dementia Cohort VUMC. The total number of correct responses was recorded and averaged over the evaluated amount of letters.

The domain information processing speed was assessed by the TMTA-A, the Stroop Color Word Test I and II, and the Digit Symbol-Coding Test (DSCT) of the WAIS-III or the Letter Digit Substitution Test (LDST). In both the DSCT and the LDST, patients were asked to copy as many symbols or digits according to a code key in a specific amount of time. The DSCT was performed in 65 patients and the LDST in 696 patients. The difference in time (60 vs 90 seconds) was resolved by the use of *Z* scores for each version in the creation of the cognitive domain.

**Table 4:** Neuropsychological test scores and cognitive domains in the study population (N=861).

Neuropsychological tests and cognitive domains				Patients, n (%)		Raw test scores, mean (SD)
**Working memory**				721 (83.7)		
	WAIS-III Digit Span forward [24]			715 (83.0)		5.4 (1.1)
	WAIS -III Digit Span backward			721 (83.7)		4.0 (1.0)
**Memory**				854 (99.2)		
	RAVLT trials 1-5 [25]			815 (94.7)		28.9 (11.5)
	VAT part A [26]			783 (90.9)		8.9 (3.7)
	RAVLT delayed recall			810 (94.1)		4.3 (3.8)
	RAVLT recognition			806 (93.6)		25.0 (4.1)
**Attention and executive functioning**				848 (98.5)		
	Ratio TMTA part B/TMT part A [27]			635 (73.8)		3.1 (1.4)
	Stroop Color Word Test III/(I and II) [28]			729 (84.7)		1.2 (0.4)
	Category fluency (Animals) [29]			833 (96.7)		15.0 (6.6)
	**Letter fluency** [29]					
		N+A	66 (7.6)		17.5 (7.8)	
		D+A+T	626 (72.7)		26.8 (13.2)	
	Information processing speed			837 (97.2)		
	TMTA part A			792 (92.0)		72.0 (55.2)
	Stroop Color Word Test I			796 (92.5)		60.3 (25.5)
	Stroop Color Word Test II			788 (91.5)		86.2 (40.6)
	**WAIS-III** [24]					
		SDMT	65 (7.5)		42.0 (15.2)	
		LDST	696 (80.8)		33.1 (12.7)	
	**Perception and construction**			705 (81.9)		
		Incomplete Letters	696 (80.8)		17.4 (4.0)	
		Dot Counting	682 (79.2)		9.3 (1.3)	

The cognitive domain perception and construction was made using the Visual Object and Space Perception Battery, administering two separate tests known as the Incomplete Letters and Dot Counting. The numbers of correct responses were recorded.

*Z* scores were created for each individual test (reversed *Z* scores for the TMT and Stroop Color Word Test). The test *Z* scores were averaged to create domain *Z* scores. If patients were unable to perform a test for various reasons, the test was defined as a missing variable. Where applicable, reports on the TRACE-VCI study will report proportions of subjects with missing values and explore potential biases. If individual test scores were missing, the domain *Z* score was based only on the available tests.

### Laboratory Testing

Plasma fasting or nonfasting glucose level, cerebrospinal fluid (CSF), and DNA for apolipoprotein E (APOE) genotyping were collected in a subset of the study population. Collection of CSF biomarkers is not a standard procedure in memory clinics in the Netherlands, but in our centers it is performed quite frequently, at the discretion of the doctor and the patient. CSF concentrations of amyloid B 1-42 (Aβ42), tau and/or total tau phosphorylated at threonine 181 (p-tau) were measured at a central laboratory for clinics at the Department of Clinical Chemistry of the VUMC in 62.8% (541/861) of patients [30]. In 51.6% (444/861) of patients, APOE genotyping was performed. For APOE genotyping, DNA was isolated from 10ml ethylenediamine tetraacetic acid blood. Subjects were classified as APOE e4 carriers if they had one or two e alleles and as noncarriers if they had no e4 alleles.

**Table 5:** Brain MRI acquisition in the study population (N=861).

	Model	Field strength (Tesla)	Patients, n (%)
**GE Medical Systems**			
	Signa HDxt	1.5	71 (8.2)
	Signa HDxt	3.0	475 (55.2)
	Discovery MR 750	3.0	73 (8.5)
**Philips Medical Systems**			
	Ingenuity	3.0	44 (5.1)
	Ingenia	3.0	152 (17.7)
	Achieva	3.0	40 (4.6)
Others^a^			6 (0.7)

^a^1.5 Tesla GE Medical Systems Signa Excite, n=1 (0.1%); 1.5 Tesla Philips Medical Systems Achieva, n=2 (0.2%); 1.5 Tesla Philip Medical Systems Intera, n=1 (0.1%); 1.5 Tesla Sonata Siemens, n=2 (0.2%).

### MRI Assessment

#### Brain MRI Scan

Brain MRI scans were performed on a 3.0 Tesla (94.1%, 810/861) or 1.5 Tesla MRI scanner (5.9%, 51/861). Most scans were performed on a GE (72.0%, 620/861) or Philips (27.8%, 239/861) MRI scanner (Table 5). The MRI scan protocol included the following sequences: 3D T1-weighted, T2-weighted, T2*-weighted/susceptibility-weighted imaging (SWI) and fluid-attenuated inversion recovery (FLAIR) sequences. A total of 850 (98.7%) patients were scanned using all of these sequences. In 11 patients (1.3%), a 2D T1-weighted sequence was acquired instead of a 3D T1-weighted sequence and/or no FLAIR sequence was available.

The MRI sequence parameters were as follows:

- 1.5 Tesla GE Signa HDxt—3D T1-weighted sequence (172 slices, voxel size: 0.98x0.98x1.50 mm^3^, Repetition Time (TR)/Echo Time (TE): 12.3/5.2 ms), 3D FLAIR sequence (128 slices, voxel size: 1.21x1.21x1.30 mm^3^, TR/TE/Inversion Time (TI): 6500/117/1987 ms), 2D T2-weighted sequence (48 slices, voxel size: 0.98x0.98x3.00 mm^3^, TR/TE: 1000/23.9 ms), 2D T2*-weighted sequence (48 slices, voxel size: 0.98x0.98x3,00 mm^3^, TR/TE: 1000/24 ms)
- 3.0 Tesla GE Signa HDxt—3D T1-weighted sequence (176 slices, voxel size: 0.94x0.94x1.00 mm^3^, TR/TE: 7.8/3.0 ms), 3D FLAIR sequence (132 slices, voxel size: 0.98x0.98x1.2 mm^3^, TR/TE/TI: 8000/126/2340), 2D T2-weighted sequence (48 slices, voxel size: 0.49x0.49x3.00 mm^3^, TR/TE/: 8610/112 ms), 3D SWI sequence (48 slices, voxel size: 0.49x0.49x3.00 mm^3^, TR/TE: 31/25 ms)
- 3.0 Tesla GE Discovery MR 750—3D T1-weigthed sequence (176 slices, voxel size: 0.94x0.94x1.00 mm^3^, TR/TE: 8.2/3.2 ms), 3D FLAIR sequence (160 slices, voxel size: 0.98x0.98x1.2 mm^3^, TR/TE/TI: 8000/130/2340 ms), 2D T2-weighted sequence (48 slices, voxel size: 0.49x0.49x3.00 mm^3^, TR/TE/: 8300/112 ms), 3D SWI sequence (44 slices, voxel size: 0.49x0.49x3.00 mm^3^, TR/TE: 31/25 ms)
- 3.0 Tesla Philips Ingenuity—3D T1-weighted sequence (180 slices, voxel size: 0.87x0.87x1.00 mm^3^, TR/TE: 9.9/4.6 ms, 3D FLAIR sequence (321 slices, voxel size: 1.04x1.04x0.56 mm^3^, TR/TE/TI: 4800/279/1650 ms), 2D T2-weighted sequence (45 slices, voxel size: 0.49x0.49x3.3 mm^3^, TR/TE: 2500-5000/100 ms, 3D SWI sequence (247 slices, voxel size: 0.43x0.43x0.60 mm^3^, TR/TE: 29x20 ms)
- 3.0 Tesla Philips Achieva and Ingenia—3D T1-weighted sequence (192 slices, voxel size: 1.00x1.00x1.00 mm^3^, TR/TE: 7.9/4.5 ms), 2D FLAIR sequence (48 slices, voxel size: 0.96x0.95x3.00 mm^3^, TR/TE/TI: 11000/125/2800 ms), 2D T2-weighted sequence (48 slices, voxel size: 0.96x0.96x3.00 mm^3^, TR/TE/TE2: 3198/140 ms), 2D T2*-weighted sequence (48 slices, voxel size: 0.96x0.96x3.00 mm^3^, TR/TE: 1653/20 ms)

#### Visual MRI Ratings

Medial temporal lobe atrophy (MTA) was visually rated (possible range of scores for each side, 0-4) on reconstructions (perpendicular to the long axis of the hippocampus) of the 3D T1-weighted images [31]. White matter hyperintensities (WMH) were rated using the Fazekas scale (WMH grade 0-3: none or a single punctate lesion, multiple punctate lesions, beginning confluence of lesions, large confluent lesions) on the FLAIR images [13]. Lacunar infarct(s), (sub)cortical infarct(s), microbleed(s), and intracerebral hemorrhage(s) were all rated in line with the STRIVE (Standards for Reporting Vascular Changes on Neuroimaging) criteria [12]. Ratings were performed by or under supervision of a neuroradiologist (in training).

#### Image Processing

Despite heterogeneity in MRI acquisition, we will obtain volumetric brain measures for all subjects. Total brain volume, white and gray matter volume, CSF volume, intracranial volume, and WMH volume will be calculated for all subjects, using the 3D T1-weighted sequences. We are currently evaluating automated image processing methods that can best accommodate differences in acquisition. Final methods will be selected based on accuracy across the different types of MRI acquisitions and robustness across field strength [32,33].

### Clinical Diagnosis

Clinical diagnoses were established at multidisciplinary consensus meetings after the 1-day memory clinic evaluation at participating centers. Diagnoses were verified by the researchers of the TRACE-VCI project based on screening of medical files. Patients were divided in three categories of severity of cognitive impairment: dementia, MCI, and NOCI. Table 6 presents the different categories.

Patients were diagnosed with dementia if there was a clear decline in cognitive function defined as a deficit in ≥2 cognitive domains at neuropsychological testing and interference in daily living [4]. Dementia was further classified due to its main etiology, based on internationally established diagnostic criteria without knowledge of CSF biomarkers or APOE genotyping results, in a vascular [34], neurodegenerative [35-37], or unknown origin (Table 6).

MCI was a clinical diagnosis defined as complaints of deterioration in cognitive function from a prior baseline and objective evidence of impairment in at least one cognitive domain. Instrumental activities of daily living were normal or only mildly impaired [4].

Finally, NOCI was defined as having cognitive complaints, but no objective cognitive impairment on neuropsychological testing. In a memory clinic setting, such patients are also referred to as having subjective complaints or subjective cognitive impairment [11].

**Table 6:** Severity of cognitive impairment and clinical diagnosis (N=861).

			Patients, n (%)
No objective cognitive impairment			198 (23.0)
Mild cognitive impairment			213 (24.7)
**Dementia**			450 (52.3)
	Vascular [34]		37 (8.2)
	**Neurodegenerative**		387 (85.8)
		Alzheimer’s disease [36]	305 (78.8)
		Frontotemporal dementia [37]	25 (6.5)
		Lewy body dementia [35]	20 (5.2)
		Others^a^	37 (9.6)
	Unknown etiology^b^		27 (6.0)

^a^Such as Primary Progressive Aphasia [38], Cortical Basal Syndrome [39], and Progressive Supranuclear Palsy [40].

^b^Dementia of unknown origin; further examination needed to state diagnosis.

### Follow-Up Investigation

#### Collection of Follow-Up Data

Follow-up data were collected during a visit at the outpatient clinic around 2 years from baseline visit. At the baseline visit, the doctor and patient decided if a follow-up visit was necessary and in the best interest of the patient. All patients who did not attend the outpatient clinic after approximately 2 years were contacted by phone and a close relative or friend was also contacted to complement the information. If patients could not provide information themselves, only close relatives of friends were interviewed. If patients were unreachable or gave no permission to contact them personally in the future, the general practitioner or doctor of the nursing home was contacted if permitted by informed consent at baseline visit.

#### Primary Outcome Measure of Prognostic Studies

The primary goal of the prognostic studies of the TRACE-VCI study is to identify which patients have a poor clinical outcome in the years following the initial visit. We therefore defined a composite primary outcome measure that is robust, clinically relevant, and could also be collected from patients who did or could not visit the outpatient clinic again after 2 years. Follow-up was not collected from patients with a low MMSE [22] score of <20 or a CDR [25] of >1 at baseline visit. This included 150 (17.4%) of all patients who were included at baseline.

For the primary prognostic analyses in the TRACE-VCI study, poor clinical outcome was defined as a composite of (1) marked cognitive decline, (2) occurrence of a major vascular event, and (3) death. Marked cognitive decline was defined as a change in CDR of ≥1 and/or institutionalization due to cognitive dysfunction during the follow-up period [41]. Occurrence of a major vascular event during follow-up was defined as a stroke, myocardial infarction, or clinical manifestations of arterial disease requiring surgical or endovascular intervention (eg, a coronary bypass operation, carotid artery stenosis, arterial dotter procedure, or stent placement).

#### Additional Follow-Up Measurements

During the outpatient follow-up visit and by telephone contact, additional follow-up information was collected. The standardized DAD questionnaire was collected from a proxy-respondent [23]. The opinion of the patient and proxy-respondent on progression, stability, or improvement of cognitive symptoms was recorded. Noncognitive outcome information collected during the outpatient clinical visit or by telephone contact included the use of a walking aid, walking distance, number of falls, and the possible related injury. Relevant changes in medical history were also recorded in the database.

The majority of patients who had an outpatient follow-up visit also underwent an extensive neuropsychological examination. Cognitive domains were recreated by the use of *Z* scores, using the same tests as on baseline visit evaluating changes in the different cognitive domains.

### Statistical Analysis

#### Sample Size Considerations

The TRACE-VCI study was designed to address several research questions. Therefore, statistical power is not a unitary construct for the study. Overall, the total cohort of 861 subjects will allow exploration of prognostic models including up to 10 predictors, at a power of 0.8, to detect small effect sizes (Cohen’s *d*=0.2 and f^2^=0.02)

#### Planned Analysis

For the first aim—establishing clinical features of different patterns of brain injury—cross-sectional analysis on baseline will be performed on all subjects. Regression analyses will be used with adjustments for age, level of education, and gender to investigate the association between vascular brain injury and cognitive and noncognitive outcomes. Because most injury types are present in >100 patients (see Table 1), we will be able to detect small to moderate effect sizes (Cohen’s *d*=0.2-0.4) between groups of patients with different lesion types (power 0.8, alpha<.05). Depending on the research questions, other covariates may be added stepwise to the model to investigate the relation further. Factor analyses will be used to investigate clusters of VCI phenotypes.

For the second aim, identification of key prognostic factors, longitudinal analysis will be performed on all patients that were eligible for follow-up (as described above). The prognostic value of a variable will be examined using Cox proportional hazard models. Accelerated cognitive decline, new major vascular events, or death are the primary outcome measures. We will start with univariate models of all possible predictors with age, level of education, and gender as covariates.

## Discussion

### Principal Considerations

The TRACE-VCI study is a large prospective cohort study evaluating the clinical features and prognosis of VCI in a memory clinic population. The majority of previous studies on vascular brain injury and cognition are either based on the general population, focusing on patients who experienced a stroke or transient ischemic attack (TIA) [7], or primarily address one particular type of vascular brain injury, such as WMH [42]. It is clearly important to document which clinical phenotypes related to vascular injury can be identified in a memory clinic setting and which factors determine prognosis for the individual patient. With regard to clinical phenotypes, it is important to know to which extent the type or location of vascular injury determines the cognitive profile. This may support a more accurate diagnosis, particularly in the context of co-occurring neurodegenerative aetiologies, which will be much more common in memory clinic patients than in other populations. The TRACE-VCI study will explore these profiles and, because of availability of CSF biomarkers in a substantial proportion of patients, will be able to explore the interaction between vascular brain injury and processes related to Alzheimer’s disease. With regard to prognosis, risk factors for cognitive dysfunction in the general population may not necessarily determine the rate of further cognitive decline among people attending a memory clinic. Identification of specific VCI phenotypes in a memory clinic setting may also support selection of patients for potential future treatment trials. The TRACE-VCI study collected this information in the context of actual clinical practice.

The operational definition of possible VCI as developed for the TRACE-VCI study should be addressed in this discussion. When the TRACE-VCI study was initiated in 2009, the construct of VCI had been introduced [2,3], but widely accepted clinical criteria for VCI were still lacking. We intentionally developed nonrestrictive criteria for our study. We chose not to define minimal thresholds for cognitive dysfunction. The rationale for this is that patients with cognitive decline as a result of vascular brain injury may not always develop cognitive deficits that are severe enough to be classified as MCI. A strict method to separate people with these more subtle cognitive changes from patients who complain, but actually have no change in cognitive performance at all, is not available. Hence, cognitive complaints are the entry criterion for the TRACE-VCI study and people without cognitive impairment are classified as NOCI, also referred to as subjective cognitive impairment [11]. We decided not to use the label subjective because this term has a connotation of nonconfirmed or even psychogenic complaints in medical practice. We did specify a minimal burden of vascular injury because the construct of VCI requires the presence of vascular brain injury. We deliberately did not include a criterion on a presumed causal relation between cognitive dysfunction and the observed vascular injury as this may in many cases rely on assumption, while the main purpose of the TRACE-VCI study is to determine if vascular brain injury really matters in memory clinic patients. Importantly, we did not exclude patients with evidence of co-occurring neurodegenerative disease as the construct of VCI also concerns the presence of vascular as well as neurodegenerative etiologies [3,4].

After the initiation of the TRACE-VCI study, diagnostic criteria for VCI have been proposed by international working groups, including criteria for Vascular Cognitive Impairment from the American Heart Association/American Stroke Association (AHA/ASA) [4] and criteria for vascular cognitive disorders from the International Society of Vascular Behavioural and Cognitive Disorders (VasCog) Society [43]. Unlike our operational VCI criteria, both the AHA/ASA and the VasCog criteria define a threshold for severity of cognitive dysfunction. When appropriate, analyses in the TRACE-VCI can be adapted to modify these criteria by excluding people with NOCI. The AHA/ASA criteria also apply to patients with evidence for co-occurring neurodegenerative or other causes of cognitive impairment, but these patients are labeled as possible VCI, similar to our operational definition of VCI. This is different from the VasCog criteria [43], which relates only to subjects with evidence for predominantly vascular etiology of cognitive impairment. The VasCog criteria consider evidence of other etiologies, including a neurodegenerative disorder, as an exclusion criterion for the diagnosis VCI. With regard to the causality of the relation between cognitive dysfunction and vascular injury, the AHA/ASA criteria distinguish between probable and possible VCI. Probable VCI is diagnosed when a clear temporal relationship between a vascular event (eg, a clinical stroke) and onset of cognitive deficits is present or if there is a clear relationship in the severity and pattern of cognitive impairment and the presence of diffuse subcortical cerebrovascular disease pathology (eg, as in cerebral autosomal dominant arteriopathy with subcortical infarcts and leukoencephalopathy). This temporal relationship is also recorded in the TRACE-VCI database, and we can apply these AHA/ASA criteria also in our dataset.

A key objective of TRACE-VCI is to identify prognostic factors in patients with VCI. To this end, we wanted to define a clinically meaningful outcome measure that we could collect in the majority of patients and also in those who could or would not revisit the clinic for follow-up. We chose a composite measure that reflects the primary poor outcome of VCI: marked cognitive decline, a major vascular event, or death. We did perform repeated cognitive assessments in patients who attended the outpatient clinic again, and we will perform analyses on these dates. However, we decided not to base our definition of marked cognitive decline on these assessments as that would induce substantial attrition.

### Strengths and Limitations

A strength of the TRACE-VCI study is that it addresses the clinical features and prognosis of VCI in a memory clinic setting. It thus fills a knowledge gap, as there are few available cohort studies of VCI in this particular setting [7]. As a consequence, despite the fact that vascular brain injury on MRI is a very common finding in memory clinic patients, uncertainty can exist about its clinical and prognostic relevance in individual cases. TRACE-VCI includes a large cohort of memory clinic patients with 2-year follow-up and detailed data on cognitive performance, imaging markers, and comorbid conditions. A potential weakness of our study is that patients were included at tertiary referral centers, which may affect generalizability of the findings. Moreover, although patients were evaluated in a standardized fashion, the study does rely on data collection in the context of clinical care. Therefore, there is some heterogeneity in data acquisition (eg, MRI protocols) and not all parameters are available for all participants. Where applicable, reports on the TRACE-VCI study will specify proportions of subjects with missing values and explore potential biases. Finally, aspects of medical history (eg, vascular events) were based on self-report, which could be affected by recall bias. Yet, in our clinics the information is verified with an informant (eg, relative) and the information provided by the referring physician. Moreover, all patients bring a medication list provided by their pharmacy, and this is also scrutinized by the treating physician to identify relevant comorbidities.

### Conclusion

The follow-up of our study is nearly complete, and data-cleaning and processing are in progress. The TRACE-VCI cohort study will provide detailed information on the phenotypes of VCI in a memory clinic setting to reveal the progression of cognitive decline and identify prognostic factors.

## Abbreviations

AHA/ASA: American Heart Association/American Stroke Association
APOE: apolipoprotein E
Aβ42: amyloid B 1-42
BMI: body mass index
CAMCOG: Cognitive and Self-Contained Part of the Cambridge Examination for Mental Disorders of the Elderly (CAMDEX)
CDR: Clinical Dementia Rating
CSF: cerebrospinal fluid
DAD: Disability Assessment for Dementia
DSCT: Digit Symbol-Coding Test
FLAIR: fluid-attenuated inversion recovery
GDS: Geriatric Depression Scale
LDST: Letter Digit Substitution Test
MCI: mild cognitive impairment
MMSE: Mini-Mental State Examination
MTA: medial temporal lobe atrophy
NOCI: no cognitive impairment
NPI: Neuropsychiatric Inventory
p-tau: total tau phosphorylated at threonine 181
RAVLT: Rey Auditory Verbal Learning Test
STRIVE: Standards for Reporting Vascular Changes on Neuroimaging
SWI: susceptibility-weighted imaging
TMT: trail making test
TR/TE/TI: Repetition Time/Echo Time/Inversion Time
TRACE-VCI: Utrecht Amsterdam clinical features and prognosis in vascular cognitive impairment
UMCU: University Medical Centre Utrecht
VasCog: International Society of Vascular Behavioural and Cognitive Disorders
VAT: Visual Association Test
VCI: vascular cognitive impairment
VUMC: VU (Vrije (Free)) University Medical Centre
WAIS-III: Wechsler Adult Intelligence Scale – 3rd edition
WMH: white matter hyperintensities
